# Cognitive correlates of attention-deficit hyperactivity disorder in children and adolescents with high intellectual ability

**DOI:** 10.1186/s11689-020-9307-8

**Published:** 2020-02-10

**Authors:** María Cadenas, Catharina Hartman, Stephen Faraone, Kevin Antshel, África Borges, Lianne Hoogeveen, Nanda Rommelse

**Affiliations:** 1 Radboud UMC, Nijmegen, The Netherlands; 2 Faculty of Health Sciences, University of La Laguna, San Cristóbal de La Laguna, Spain; 3 University Medical Center Groningen, University of Groningen, Groningen, The Netherlands; 4 State University of New York Upstate Medical University, Syracuse, NY USA; 5 K.G. Jebsen Centre for Research on Neuropsychiatric Disorders, University of Bergen, Bergen, Norway; 6 Department of Psychology, Syracuse University, Syracuse, NY USA; 7 Faculty of Social Sciences, Radboud University Nijmegen, Nijmegen, The Netherlands

**Keywords:** ADHD, High intelligence, Giftedness, Twice exceptional, Cognition

## Abstract

**Background:**

There is an ongoing debate as to whether attention-deficit hyperactivity disorder (ADHD) in highly intelligent individuals has a similar presentation as in average intelligent individuals. The aim of this study was to examine the cognitive correlates of ADHD in highly intelligent children and adolescents with ADHD.

**Method:**

Two independent samples (*N* = 204 and *N* = 84) of (1) high intelligence quotient (IQ) (IQ ≥ 120) children and adolescents with ADHD were used, carefully matched on age, gender, ADHD severity, and IQ with (2) control participants with high intelligence, (3) participants with ADHD with an average intelligence (IQ 90–110), and (4) control participants with an average intelligence. These samples were selected from the Dutch node of the International Multicenter ADHD Genetics (NeuroIMAGE) and Tracking Adolescents’ Individual Lives Survey (TRAILS) cohorts, respectively, in which a large battery of cognitive tasks was administered. Linear mixed models were used to examine the main effects of ADHD and IQ and their interaction on cognitive performance.

**Results:**

ADHD-control group differences were not moderated by IQ; mostly equally large ADHD-control differences in cognitive performance were found for high versus average intelligent groups. The small moderating effects found mostly indicated somewhat milder cognitive problems in highly intelligent individuals with ADHD. Overall, highly intelligent children and adolescents with ADHD performed at the level of the average intelligent control children.

**Conclusions:**

Our findings indicate the cognitive profile of ADHD is similar in highly versus average intelligent individuals with ADHD, although ADHD-related cognitive deficits may be easily overlooked in the high intelligence population when compared to the typical (i.e., average intelligent) control group.

## Background

Attention-deficit hyperactivity disorder (ADHD) is defined by high levels of inattention, impulsiveness, and/or hyperactivity that significantly impair daily functioning and are pervasive across situations [1]. A strong body of literature describes the cognitive correlates of ADHD. A wide range of cognitive domains—from basic reaction time variability to complex executive and social cognitive functions—are on average impaired in individuals with ADHD [2–6]. However, there is an ongoing debate as to whether these cognitive domains are equally impaired in individuals with ADHD and high intellectual capacity [7–9]. Surprisingly, very few studies have examined this issue directly and no previous study has used both ADHD-matched and IQ-matched control groups to investigate this research question. The innovative aim of this study was therefore to determine the cognitive profile of individuals with ADHD and high intelligence using an individually matched four-group design with both average and highly intelligent individuals with and without ADHD.

**Fig. 1:**
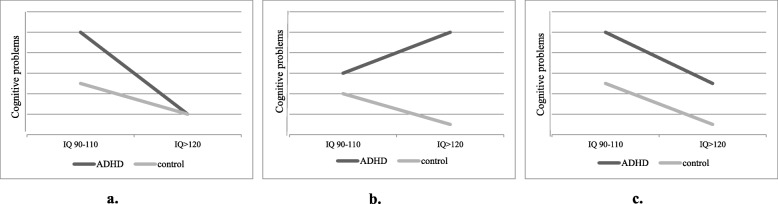
**a** The *mimicing-hypothesis*. According to this hypothesis, individuals with high intelligence levels show high levels of activity, attention difficulties, and impulsivity that are not thought to be indicative of ADHD, but rather a consequence of the very fast processing style inherent to a high intelligence level. Highly intelligent individuals with ADHD symptoms would not show the cognitive correlates that are usually found in (average intelligent) individuals with ADHD. **b** The *strongly atypical-hypothesis*. A high intelligence level is (strongly) protective against ADHD. If ADHD symptoms are present in highly intelligent individuals, they mark a severe form of the disorder given the rarity of these symptoms in this population. As a consequence, a relatively more severe cognitive profile may be present. **c** The *compensating-hypothesis*. The cognitive correlates of highly intelligent individuals with ADHD are similar in type and severity as those in average intelligent individuals with ADHD but are easily overlooked when compared with average intelligent controls. According to this hypothesis, the cognitive correlates of ADHD in highly intelligent individuals are only observed when compared with highly intelligent controls, but not—or less so—when compared with the average

Previously, it has been argued that high intelligence “mimics” ADHD [10, 11]. According to this hypothesis, individuals with high intelligence frequently show high levels of activity, attention difficulties, and problems following rules and with task persistence [10–13]. Individuals with high intelligence can also achieve at a level lower than expected considering their cognitive abilities and experience social difficulties [14, 15]. These characteristics resemble those of individuals with ADHD, but are not thought to be indicative of ADHD in these highly intelligent individuals, but rather a consequence of their very fast processing style and mismatch with their environments that are tailored for average intelligent individuals (i.e., and thus understimulating for highly intelligent individuals). As a result, several authors have warned about the danger of misdiagnosis or overdiagnosis of ADHD in the highly intelligent population [10, 16, 17]. Based on the hypothesis that high intelligence may mimic ADHD without the “true” disorder being present, it can be hypothesized that highly intelligent individuals with ADHD symptoms will not show the cognitive impairments that are usually found in (average intelligent) individuals with ADHD (Fig. 1a: “mimicing-hypothesis”).

In contrast, in a recent systematic review on the relationship between intelligence and ADHD, high intelligence level was found to be strongly protective against ADHD symptoms and related cognitive problems [18]. Results indicated children and adults with a high intelligence quotient (IQ) had low levels of ADHD symptoms and a low chance of having the cognitive problems that are often found in individuals with ADHD [18]*.* These conclusions were further supported in a large population-based sample where intelligence levels were inversely related to ADHD (and comorbid) symptoms [19]. Moreover, if ADHD symptoms were present in children with a high IQ, these symptoms were associated with the same amount of impairment in school functioning as in children with an average IQ, arguing against the idea that these symptoms are harmless “mimics” of ADHD. Based on all of the above, it can be hypothesized that if ADHD symptoms are present in highly intelligent individuals, the ADHD symptoms mark a severe form of the disorder given the rarity of these symptoms in this population. As a consequence, a relatively more severe cognitive profile may be present (Fig. 1b: “strongly atypical-hypothesis”).

Finally, it has been argued that the cognitive correlates of highly intelligent individuals with ADHD are similar in type and severity as those in average intelligent individuals with ADHD [7, 20] but are easily overlooked when compared to typical (i.e., average intelligent) controls [2]. According to this hypothesis, when ADHD and high intelligence occur together, the high intelligence may compensate/mask the ADHD-related impairments [16, 20–23]. As a consequence, the cognitive correlates of ADHD in highly intelligent individuals are only observed when compared to highly intelligent controls, yet not when compared to average intelligent controls (Fig. 1c: “compensating-hypothesis”). ADHD-related problems (such as underachievement at school) may thus be easily overlooked when compared to the typical (average intelligent) classmate [2, 23].

The only way to examine these three hypotheses is by using a four-group design, with both average and highly intelligent ADHD cases and controls, to examine whether group differences are equally large in average versus highly intelligent groups. To the best of our knowledge, no prior study has used this design. The few studies that have examined the cognitive correlates of highly intelligent individuals with ADHD mostly compared highly intelligent ADHD cases with highly intelligent controls, sometimes in combination to population norms [2, 7, 9, 22, 24]. Combining the findi4ngs of these studies, it can tentatively be concluded that findings are mostly in line with the “compensating-hypothesis” although more robust data are needed to draw firm conclusions.

The aim of this study was therefore to examine the cognitive correlates of ADHD in highly intelligent individuals with ADHD using a carefully individually matched four-group design consisting of average intelligent (IQ 90–110) and highly intelligent (IQ ≥ 120) ADHD cases and controls. The cutoff point of an IQ ≥ 120 is in line with previous studies on this topic [7, 8, 24] and represents less than 10% of the general population. Participants were further individually matched for age and gender to rule out the effect of potential confounders. Two independent cohorts were available, a clinical sample (*N* = 204) and a population-based sample (*N* = 84). In both cohorts, a wide range of cognitive parameters was available, including response inhibition, verbal working memory, timing variability, sustained attention, and social cognition. The key question that was examined was whether a diagnosis by IQ interaction was present. If the *mimicing symptoms-hypothesis* was supported, an interaction effect was expected where ADHD-control differences would only be found in average intelligent but not in highly intelligent participants. If the *strongly atypical-hypothesis* was confirmed, an interaction effect was expected where ADHD-control differences would be larger in highly intelligent participants. Finally, if the *compensating-hypothesis* was confirmed, no interaction effect was expected and ADHD-control differences would be equally large in average versus highly intelligent groups.

## Methods

### Participants

#### Cohort 1

The sample originated from the Dutch node of the International Multicenter ADHD Genetics (IMAGE) study (www.neuroimage.nl), a longitudinal sample with three measurement waves between 2003 and 2015 (wave 1, 2003–2006; wave 2, 2009–2012; wave 3, 2013–2015). The NeuroIMAGE cohort consists of probands with ADHD, their biological parents, and full biological siblings. Participants were usually assessed in more than one wave. The description of the sample, the measurements, and the recruitment procedure have been described in detail in previous papers [25]. A total of 51 (1) individuals with ADHD and an IQ ≥ 120 were successfully individually matched in quartets based on age (maximum of 1 year difference within each quartet) and gender to (2) controls with an IQ ≥ 120, (3) individuals with ADHD and an IQ 90–110, and (4) controls with an IQ 90–110. Controls did not meet the ADHD criteria and had no first-degree family member with a suspected or known ADHD diagnosis. Further, ADHD subtype was used to further match, as close as possible, the average and high IQ ADHD groups regarding ADHD severity. IQ was used to match cases and controls in the highly intelligent groups and in the average intelligent groups, with a maximum of 5 IQ-point difference between individuals within one quartet. This resulted in *N* = 204 assessments in total, of which *n* = 140 represented unique participants. Repeated assessments included *n* = 20 participants with data collected in two different waves/time points (i.e., participant was of a different age during both assessments; *n* = 40 data points); *n* = 1 participant was measured in two waves and within one of those waves in two quartets (*n* = 3 data points). In addition, data was duplicated for *n* = 6 and triplicated for *n* = 3 participants, respectively (not participants with ADHD and an IQ ≥ 120), to be used for individual matching purposes when no other participants fulfilled the strict matching criteria (*n* = 21 data points). To correct for the relatedness of measurements between waves for one participant as well as for duplication of data, sensitivity analyses were performed excluding these extra data points. For more information, see Table 1.

**Table 1:** Sample description

	ADHD + IQ ≥ 120		Control + IQ ≥ 120		ADHD + IQ 90–110		Control + IQ 90–110		*χ*^*2*^ Student’s *t* test *F*
	*N*	M (SD)/%	*N*	M (SD)/%	*N*	M (SD)/%	*N*	M (SD)/%	
Cohort 1									
ADHD diagnosis									
Inattentive	21	41.2			17	33.3			n.s
Hyperactive	5	9.8			7	13.7			
Combined	22	43.1			25	49			
Unspecified	3	5.9			2	3.9			
IQ score	51	126.2 (5.1)	51	126.9 (5.5)	51	101.3 (4.9)	51	101.5 (4.3)	n.s*
Age in years	51	15.4 (4.5)	51	15.1 (4.4)	51	15.4 (4.5)	51	15.3 (4.4)	n.s
Gender									
Male	29	56.9	29	56.9	29	56.9	29	56.9	n.s
Cohort 2									
ADHD diagnosis									
Inattentive	10	47.7			10	47.7			n.s
Hyperactive	3	14.3			3	14.3			
Combined	8	38.1			8	38.1			
IQ score	21	124 (3.2)	21	124 (3.2)	21	97.2 (4.2)	21	97.2 (4.2)	n.s*
Age in years	21	11.18 (0.6)	21	11.11 (0.42)	21	11.15 (0.42)	21	11.20 (0.48)	n.s
Gender									
Male	17	81	17	81	17	81	17	81	n.s

n.s* no significance between IQ-matched groups

#### Cohort 2

This sample originated from the Tracking Adolescents’ Individual Lives Survey (TRAILS), a Dutch cohort study following 2230 children from the general population and 540 children referred to an outpatient clinic before the age of 11, from early adolescence (age range 10–12) to young adulthood (www.trails.nl). The recruitment procedure has been described in other papers (see [19, 26]). Briefly, participants were recruited from the general population in the northern part of the Netherlands, including both urban and rural areas. In addition to the population-based cohort, the clinical cohort was recruited in parallel in a child psychiatry center with the same catchment area as the population sample. For the current study, data from wave 1 was used. A total of *n* = 21 highly intelligent participants with ADHD were available, of whom *n* = 2 had a likely-ADHD diagnosis (based on questionnaire data only, see measures below) originating from the population-based cohort and *n* = 19 had a confirmed ADHD diagnosis based on structured diagnostic interview originating from the clinical cohort. The quantitative ADHD symptom severity scores of these *n* = 2 participants (*M* = 12) were at the same average score of the *n* = 19 participants with a confirmed ADHD diagnosis (*M* = 11.78), indicating ADHD symptom severity was comparable. These participants were matched in the same manner as described for participants in cohort 1, resulting in 21 quartets, comprising *N* = 84 unique participants (see Table 1).

### Measures

#### ADHD assessment

##### Cohort 1

The Conners long-version parent and teacher questionnaires [27, 28] (youth < 18 years) or parent and self-report (youth ≥ 18 years) were used to assess ADHD symptoms. *T*-scores ≥ 63 on the Conners or DSM-IV ADHD subscales inattention, hyperactivity/impulsivity, and/or total symptoms were considered clinically significant. Those participants who scored clinically on any of these subscales were administered the Parental Account of Children’s Symptoms (PACS) [29] (wave 1) or the Schedule for Affective Disorders and Schizophrenia for School-Age Children-Present and Lifetime Version (K-SADS) [30] (waves 2 and 3). ADHD subtypes (combined, predominantly inattentive, or hyperactive/impulsive) were established according to DSM-IV-TR criteria (waves 1 and 2) or DSM-5 criteria (wave 3) (for full description of diagnostic procedures, see [25]). The majority of the cases were diagnosed with the combined type (43.1%) followed by the inattentive (41.2%), hyperactive-impulsive (9.8%), and unspecified (5.9%) subtypes, (see Table 1).

##### Cohort 2

For the assessment of ADHD, the Diagnostic Interview Schedule for Children (DISC-IV) was used [31]. In the clinical cohort, 19 out of 21 cases were diagnosed with DISC-IV. For 2 of the participants, the Dutch translations of the parent-reported Child Behavior Checklist (CBCL/6–18) and a short version of the Teacher Report Form (TRF; short version TPC) were used [26, 32]. An age- and gender-based clinical score on the parent CBCL-Attention Problem subscale in combination with a clinical teacher rating was used to define “likely-ADHD” cases in the population-based cohort. Both questionnaires have been found to be valid and reliable [26]. The majority of the participants with ADHD diagnosis had the inattentive type (47.7%), followed by the combined type (38.1%) or hyperactive-impulsive type (14.3%) (see Table 1).

#### Intelligence quotient

##### Cohort 1

Full-scale IQ of all youth was estimated using four subtests (vocabulary, similarities, block design, and picture completion) of the Wechsler Intelligence Scale for Children or Wechsler Adult Intelligence Scale-III (WISC/WAIS-III) [33, 34]. These four subtests correlate between 0.90 and 0.95 with the full-scale IQ [35].

##### Cohort 2

Full-scale IQ was estimated using two subtests (vocabulary and block design) of the WISC/WAIS-III [33, 34].

#### Cognitive assessment

##### Cohort 1

The cognitive tasks used on this sample have been described in previous papers [36, 37]. The tasks and dependent measures used are described in Additional file 1: Table S1. Briefly, six cognitive domains were assessed for this cohort: motor inhibition, verbal working memory, timing variability, motor coordination, time estimation ability, and motor speed. These domains were selected due to their theoretical relevance to ADHD.

##### Cohort 2

Complete description of the cognitive assessment is available in previous papers [38]. The tasks used for the current study are described in Additional file 1: Table S1. Briefly, the domains measured include timing variability, sustained and shifting attention, pattern recognition, and working memory.

### Procedure

Cognitive assessment took place in a quiet room, and small breaks were provided. Psychostimulants were discontinued for at least 48 h before testing took place. Both studies had medical/ethical approval.

### Statistical analyses

In both cohorts, analyses were separately carried out using the Statistical Package for the Social Sciences (SPSS) version 23. The percentage of missing data for the cognitive assessments ranged between 0 and 23% for the dependent variables for cohort 1 and between 0 and 2% for cohort 2. Missing data were replaced by using the Estimation Maximization procedure [39]. Analyses were carried out with and without imputed data; results were presented with imputed data. Variables were successfully normalized and standardized by applying a Van der Waerden transformation. Linear mixed models were used for the analyses. The linear mixed model expands the general linear model so that the data are permitted to exhibit correlated variability. This model allows for the investigation of group differences while correcting for the non-independence of data (i.e., in cohort 1, some children were included more than once, which resulted in related measurements within groups). Factors included were ADHD diagnosis (yes/no) and IQ ≥ 120 (yes/no) and their interaction to examine the potentially moderating effect of IQ on ADHD-control group differences in the manifestation of the cognitive symptoms associated with ADHD. Since groups were matched on age and gender, both ADHD groups were matched on ADHD subtype, and the IQ groups were matched on IQ; there was no need to include these variables as possible confounders. Post hoc *t* tests compared the performance of the group with ADHD and high intelligence with the control groups with average IQ and high intelligence, in order to illustrate the effect of comparing highly intelligent individuals with ADHD with IQ-matched versus average intelligence controls. Lastly, in order to consider the overall pattern of results, a principal component analysis was carried out to create a single composite score of cognitive performance. Correction for false discovery rates (FDR) for multiple testing was carried out.

## Results

### Cognitive correlates in relation to ADHD and high intelligence

No significant interaction effects were found (Additional file 2: Table S2), indicating that ADHD-control differences were similar for average and highly intelligent participants. With correction (leaving only effects of at least medium effect size), 4 of 26 main effects of ADHD (4 of 12 variables in cohort 1; 0 of 14 variables in cohort 2) and 8 of 26 main effects of IQ remained significant (2 of 12 variables in cohort 1; 6 of 14 variables in cohort 2) (see Additional file 2: Table S2).

### “Masked” cognitive problems in highly intelligent individuals with ADHD compared to typical controls

Comparing individuals with ADHD and a high intelligence to “typical” average intelligent controls, significant differences were found between highly intelligent individuals with ADHD and average intelligent controls on 2 of 26 variables, with individuals with high IQ and ADHD performing better than average intelligent controls (see Additional file 2: Table S2).

### Results for an aggregated score of cognitive performance

A principal component analysis was carried out in both samples to aggregate data across the individual cognitive parameters, in order to examine the main and interaction effects on these underlying components for summary and illustration purposes. In both samples, one main component was obtained explaining 40% of the variance in cohort 1 and 35% of the variance in cohort 2, with both speed and accuracy measures loading on this component. In cohort 1, 14 of 16 variables loaded with a weight above .30 on this factor. In cohort 2, 10 of 14 variables loaded with a weight above .30 on this factor.

**Fig. 2:**
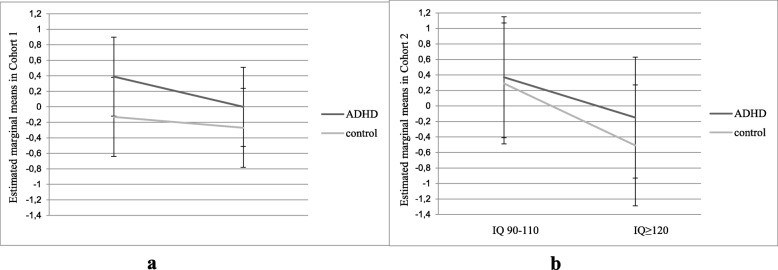
**a**, **b** Aggregated score of cognitive performance. Estimated marginal means for an aggregated cognitive score in two independent cohorts depicting individuals with ADHD and a high intelligence (cohort 1: *N* = 51, cohort 2: *N* = 21) individually matched to highly intelligent controls, average intelligent participants with ADHD, and average intelligent controls based on ADHD subtype, IQ, age, and gender. Higher scores indicate a poorer performance. No interaction effect between ADHD and IQ was found in both cohorts. Error bars represent 95% confidence intervals

Linear mixed models on the two aggregated scores revealed no significant and no trend significant ADHD by IQ interaction effects (*F* (1, 200) = 0.87, *p* = 0.35; *F* (1, 80) = 0.49, *p* = 0.49) (Fig. 2a, b). In cohort 1, a significant main effect of ADHD (*F* (1, 200) = 8.71, *p* < 0.01, *d* = 0.58) and IQ (*F* (1, 200) = 3.81, *p* < 0.05, *d* = 0.38) was found. In cohort 2, only a significant main effect of IQ was found (*F* (1, 80) = 10.35, *p* < 0.01, *d* = 0.97). Post hoc *t* tests indicated that the highly intelligent group with ADHD performed at the same level as the average intelligent control group (cohort 1: *t* = 0.69, *p* = 0.48; cohort 2: *t* = − 1.43, *p* = 0.16).

### Sensitivity analyses

Additional analyses were carried out in the NeuroIMAGE cohort in order to control for the effect of repeated assessments. As a first step, for participants who had repeated assessments in different waves of the cohort, only the first assessment was kept for analyses. This resulted in *n* = 170 participants (140 assessed-once participants plus the first assessment from *n* = 30 participants with repeated assessments). Principal component analysis and an ANOVA were carried out. Results remained similar to the original analyses, with one main component explaining around the 40% of the variance and a significant main effect for ADHD only (*F* (1, 166) = 8.62, *p* < 0.01, *d* = 0.58) but not for IQ (*F* (1, 116) = 4.64, *p =* 0.32) or an ADHD × IQ interaction (*F* (1, 166) = 0.34, *p* = 0.54). The same procedure was carried out using only the *n* = 140 unique participants. The main results remained identical, with one main component explaining 42% of the variance and no ADHD × IQ interaction was found (*F* (1, 136) = 0.05, *p =* 0.81).

## Discussion

The aim of this study was to examine the cognitive correlates of ADHD in highly intelligent children and adolescents with ADHD. Two independent cohorts were used, comprising of (1) 51 and 21 highly intelligent (IQ ≥ 120) individuals with ADHD, respectively, carefully matched on age, gender, ADHD subtype, and IQ with (2) control participants with high intelligence, (3) participants with ADHD with average intelligence (IQ 90–110), and (4) control participants with average intelligence. In both cohorts, a battery of cognitive tasks was administered. Results indicate that ADHD-control group differences were not moderated by IQ; similarly, large ADHD-control cognitive performance differences were found in high versus average intelligent groups. The small moderating effects that were found when multiple testing was not corrected for mostly indicated somewhat milder cognitive problems in highly intelligent individuals with ADHD. However, on several tasks, a moderate to large effect of IQ was found: highly intelligent participants (regardless of having ADHD or not) performed better than average intelligence participants. Highly intelligent individuals with ADHD generally performed at the level of average intelligence controls.

Results primarily support the *compensating-hypothesis* where ADHD-related deficits are compensated by the high intelligence of highly intelligent individuals with ADHD (Fig. 1c). These data are consistent with the findings of previous work regarding the cognitive correlates of highly intelligent individuals with ADHD, although those studies mostly provided indirect support since none explicitly tested the interaction between ADHD and IQ [2, 7, 22, 24]. In a broader context, these results support the validity of the ADHD diagnosis in highly intelligent individuals, a conclusion that others have similarly reached [2, 7, 9, 14, 16, 20, 22, 40].

These results also have direct clinical implications. First, and foremost, the cognitive correlates of ADHD in highly intelligent children are overall similar to those found in average intelligent children with ADHD, suggesting that similar cognitive domains can be targeted for psychodiagnostic clinical practice. However, as is the case for average intelligent participants with ADHD [6, 41, 42], effect sizes were usually small to moderate, suggesting a similar cognitive heterogeneity underlying ADHD in highly intelligent versus average intelligent populations with ADHD. Cognitive test performance can—regardless of intelligence level—never be used to confirm or refute the presence of an ADHD diagnosis. However, particular caution is needed when applying standard normed scores to the cognitive test performance of highly intelligent individuals with ADHD. Our results suggest that relative cognitive weaknesses of highly intelligent individuals with ADHD are likely not picked up using normative scores that were standardized on generally average intelligent participants. However, other studies have indicated that the relation between intelligence and executive functioning is small to moderate, suggesting IQ-based norms may not turn out to be very different from normative scores standardized on the generally average intelligent population [43]. It would be clinically very relevant if future studies could tackle this issue and quantify the risk of underestimating certain cognitive weaknesses (i.e., attention, working memory, executive functions) in highly intelligent individuals with ADHD applying standardized norms versus IQ-stratified norms.

A lingering question, which cannot be answered based on our data, is whether the high intellectual capacity of individuals with ADHD is more often overlooked relative to their typically developing highly intelligent individuals without ADHD. Given that the cognitive performance of highly intelligent youth with ADHD is similar to average IQ youth without ADHD, it is possible that peers and adults perceive these high IQ youth with ADHD differently. Overlooking high intellectual abilities in ADHD may prohibit full potential being realized. On this line, routine screening of intellectual abilities in clinical practice might add to the quality of care for this patient group (as well as to the patient group with borderline intellectual functioning, a frequently failed to be detected group). Even then, however, the ADHD symptoms might affect the working attitude and possibly lead to an IQ score that does not represent the intellectual abilities of the testee. The psychologist needs to have knowledge about ADHD as well as knowledge about possible problems caused by giftedness to be able to interpret the data.

This study is the first to use an individually matched four-group design to examine the cognitive correlates of ADHD in highly intelligent individuals. Limitations of the study are the method for measuring intelligence. IQ was estimated based on two (cohort 2) or four (cohort 1) subtests only. However, the subtests used are known to highly correlate with the full-scale IQ [40] and suitable for screening purposes in clinical practice where a full-scale IQ is too expensive and not always needed. In addition, the abbreviated IQ assessment did not include the working memory and processing speed subtests, which would have likely differentially impacted individuals with ADHD who tend to perform more poorly in these domains. Future research should consider how administering a full IQ battery may affect these results. Another limitation is the operationalization of high intelligence used in this study (IQ > 120). Using an IQ ≥ 130, a cutoff also often applied to study subjects with “high intellectual abilities” or “giftedness” (see for example [2, 11, 14]) may result in different findings. However, the IQ ≥ 120 cutoff is consistent with many earlier studies on this topic [7, 14, 18] facilitating comparison of results.

## Conclusions

In summary, our findings indicate the cognitive profile of ADHD is similar in highly versus average intelligent individuals with ADHD, although ADHD-related cognitive deficits may be easily overlooked in the high intelligence population when compared to the typical (i.e., average intelligent) control group.

## Supplementary information

**Additional file 1: Table S1.** Description of cognitive assessment.

**Additional file 2: Table S2.** Results of Cognitive correlates in relation to ADHD and high intelligence.

## Data Availability

The datasets used and/or analyzed during the current study are available from the corresponding author.

## Abbreviations

ADHD: Attention-deficit hyperactivity disorder
ANOVA: Analysis of variance
CBCL: Child Behavior Checklist
DISC: Diagnostic Interview Schedule for Children
DSM: Diagnostic and statistical manual of mental disorders
FDR: False discovery rates
IQ: Intelligence quotient
K-SADS: Affective Disorders and Schizophrenia for School-Age Children-Present and Lifetime Version
NeuroIMAGE: International Multicenter ADHD Genetics (IMAGE)
PACS: Parental Account of Children’s Symptoms
SPSS: Statistical Package for the Social Sciences
TRAILS: Tracking Adolescents’ Individual Lives Survey
TRF: Teacher Report Form
WISC/WAIS: Wechsler Intelligence Scale for Children or Wechsler Adult Intelligence Scale
