# Supplementary material for: Cognitive correlates of attention-deficit hyperactivity disorder in children and adolescents with high intellectual ability
Source: J Neurodev Disord. 2020 Feb 10;12:6. doi: 10.1186/s11689-020-9307-8 (PMC7008522; doi:10.1186/s11689-020-9307-8)
Supplement: Supplementary file 1 — Additional file 1: Table S1. Description of cognitive assessment. [file 11689_2020_9307_MOESM1_ESM.docx]

**Cognitive correlates of Attention Deficit Hyperactivity Disorder in children and adolescents with high intellectual capacity.**

**Additional file 1: Cognitive assessment.**

**Cohort 1**: The cognitive tasks used on this sample have been described in previous papers (for more information consult^1,2,3^). Briefly, for the assessment of Motor inhibition *Stop Task* was used. This task is based on the paradigm of Logan^4^ and has been used in previous studies (see for example^5,6^). The subtest *Digit span maximum span forwards and backwards^7,8^* was used to evaluate Verbal Working Memory. Two tasks from the Amsterdam Neuropsychological Tasks battery (ANT^9^) were used to assess Timing stability and Motor coordination, *Baseline speed* and *Pursuit* tasks. Finally, two tasks designed to measure Time estimation ability and Motor speed were included, *Time test* and *Motor timing^10^*. A description of the tasks selected is shown in Table S1.

**Cohort 2:** Complete description of the cognitive assessment is available in previous papers (for more information see^11,12^). Briefly, seven tasks from the ANT battery^9^ were used, *Baseline speed* (as in Cohort 1), *Sustained attention dots*, *Shifting attentional set*, *Feature identification*, *Face recognition, Identification of emotions* and *Memory search letters.* A description of the tasks selected is shown in Table S1.

**Table S1. Description of cognitive assessment.**

| ***COHORT 1*** |  |  |
| --- | --- | --- |
| **Domain and name of the task** | **Aim and description** | **Outcome measured** |
| **Inhibition** |  |  |
| Stop Task | **Motor inhibition of an ongoing response.** An object was presented to the participants which was either pointing to the right or to the left. Participants were required to press a response button that corresponded to the direction of the stimulus as fast and precisely as they could. For the stop-signal children were required to withhold their response. | Latency of the stop-process through three parameters: mean reaction time, variability and stop signal reaction time. |
| **Verbal working memory** |  |  |
| Digit span: maximum span forwards and backwards | **Verbal working memory.** Forward: the participants need to repeat a sequence of numbers in the same order. Backward: repeating the numbers in the opposite order. | Number of sequences memorized in the correct order. |
| **Timing variability** |  |  |
| Baseline speed left and right hand | **Motor speed and variability in response to an external sign.** A fixation cross in the centre of a computer screen changed unpredictably into a white square. Participants were required to press a key when the white square appeared. Same task was carried out with both hands. | Mean reaction time (ms) across hands. |
| **Motor coordination** |  |  |
| Pursuit: deviation left and right hand | **Motor control.** The task consisted in catching an object which was in constant movement using the cursor of a mouse. Participants were asked to “catch” the object precisely by putting the cursor of the mouse on the top of the object. The task was carried out first with the non-preferred hand and after with the preferred one. | Precision measured in terms of mean distance between the cursor and the object and reaction time. |
| **Time estimation ability** |  |  |
| Time test percentage absolute discrepancy 4; 8; 12; 16; 20 sec | **Time reproduction.** Stimuli with two different modalities, visual and auditive, were presented to the participants in randomly intervals of 4, 8, 12, 16 and 20 sec. Participants were required to press a button to reproduce the stimuli. | Precision of the reproduction, calculated by the discrepancy between the length of the response and the stimuli across modalities. |
| **Motor speed** |  |  |
| Motor timing | **Accuracy and variability of motor timing.** The participants were asked to press a button when they thought an interval of 1 second had passed. The start of the interval was announced by a tone. Visual feedback was given, indicating whether the response was correct, too short or too long. | Variability in terms of median and *SD* reaction time. |
| ***COHORT 2*** |  |  |
| **Timing variability** |  |  |
| Baseline speed left and right hand | Same as in Cohort 1 | Same as in Cohort 1 |
| **Sustained attention** |  |  |
| Sustained attention dots: mean reaction time hits | **Discrimination of patterns.** A total of 600 pictures with 3, 4 or 5 dots were showed during the task. Participants needed to identify pictures with 4 dots pressing a button with the finger of the preferred hand to say “yes” and with the finger of the non-preferred hand to answer “no.” Feedback was given through a noise every time the participant made a mistake. | Sustained attention was measured by mean reaction time and proportion of misses. |
| **Shifting Attentional set** |  |  |
| Shifting set | **Motor inhibition and cognitive flexibility.** Stimuli consisted of a horizontal grey bar with green or red squares moving randomly. The task was divided in three parts. In the first one, 40 trails, the moving square was green and participants were asked to press the button of the mouse which was in the same direction as the square; left button, left direction and right button for right direction. In second part, 40 trails, the square was red and participants were informed to press the button which was on the opposite direction of the square; left when it was going to the right, and right when it was going to the left. The third part, 80 trails, was a combination of the first two with direction and colour of the square changing randomly. | Mean reaction time and percentage errors across blocks. |
| **Pattern recognition** |  |  |
| Feature identification | **Recognition of abstract visuo-spatial patterns**. Target pattern was showed to the participants during the instructions. Afterwards, they were requested to identify it among others patterns by pressing a button with the preferred hand to answer “yes” or with the non-preferred hand to say “no.” Four different patterns were presented in each trial.  In total, 80 trials were showed: in 40 of them the target pattern was presented, in 20 similar non target patterns, and in the other 20 dissimilar patterns were showed. | Two indexes were measured: difference on reaction time and percentage of false alarms on similar non target patterns and dissimilar patterns. |
| **Working memory** |  |  |
| Memory search letters | **Memory search capacity.** The task was divided in three parts were pictures with four letters were presented. In the first part, 40 trails were presented were participants needed to identify letter “K”; in the second part, composed by 72 trails, participants needed to detect letters “K” and “R”; in the third part, 96 trails, letters “K”, “R” and “S” needed to be identified. Participants pressed a button with the finger of the preferred hand to say “yes” and with the non-preferred hand to say “no.” In each part, half of trails contented the target letters. | Performance was measured by the difference of reaction time in average for the three parts and the number of misses in average across the three blocks. |

**References**

| 1. Groenman AP, Oosterlaan J, Greven CU et al. Neurocognitive predictors of substance use disorders and nicotine dependence in ADHD probands, their unaffected siblings, and controls: a 4-year prospective follow-up. J Child Psychol Psychiatry.2015; 56(5):521-529. |
| --- |
| 2. van Lieshout M, Luman M, Schweren LJS et al. The Course of Neurocognitive Functioning and Prediction of Behavioral Outcome of ADHD Affected and Unaffected Siblings. J Abnorm Child Psychol. 2019 Mar;47(3):405-419. doi: 10.1007/s10802-018-0449-z. |
| 3. Rommelse, NNJ et al. Neuropsychological measures probably facilitate heritability research of ADHD. Arch Clin Neuropsychol. 2008; 23(5):579-591. |
| 4. Logan GD. On the ability to inhibit thought and action: a users’ guide to the stop signal paradigm. In: Dagenbach, D., T.H. Carr, TH, eds. Inhibitory processes in attention, memory, and language. San Diego: Academic Press; 1994:189-239. |
| 5. Alderson, R.M., Rapport, M.D. & Kofler, M.J. J Abnorm Child Psychol (2007) 35: 745. |
| 6. Oosterlaan J, Logan GD, Sergeant JA. Response inhibition in AD/HD, CD, comorbid AD/HD+ CD, anxious, and control children: A meta-analysis of studies with the stop task. The Journal of Child Psychology and Psychiatry and Allied Disciplines, 1998; 39(3):411-425. |
| 7. Wechsler D. WAIS-III Nederlandstalige bewerking. Technische handleiding. The Psychological Corporation: London; 2000 |
| 8. Wechsler D. WISC-III Handleiding. The Psychological Corporation: London; 2002. |
| 9. De Sonneville LMJ. Amsterdam Neuropsychological Task: a computer-aided assessment program. In: Den Brinker BPLM., Beek PJ, Brand AN, Maarse SJ, Mulder LJM, eds. Cognitive ergonomics, clinical assessment and computer-assisted learning: Computers in psychology, Vol. 6. Lisse, The Netherlands: Swets & Zeitlinger; 1999:204-217. |
| 10. van Meel CS, Oosterlaan J, Heslenfeld DJ,Sergeant JA. Motivational effects on motor timing in ADHD. J Am Acad Child Adolesc Psychiatry, 2005; 44:451-460. |
| 11. Boelema SR, Harakeh Z, Ormel J, Hartman CA, Vollebergh WA, van Zandvoort MJ. Executive functioning shows differential maturation from early to late adolescence: Longitudinal findings from a TRAILS study. Neuropsychology. 2014; 28(2):177. |
| 12. Brunnekreef J, De Sonneville LM, Althaus M et al. Information processing profiles of internalizing and externalizing behavior problems: Evidence from a population‐based sample of preadolescents. J Child Psychol Psychiatry. 2007;48(2):185-193. |
