# Supplementary material for: Cognitive correlates of attention-deficit hyperactivity disorder in children and adolescents with high intellectual ability
Source: J Neurodev Disord. 2020 Feb 10;12:6. doi: 10.1186/s11689-020-9307-8 (PMC7008522; doi:10.1186/s11689-020-9307-8)
Supplement: Supplementary file 2 — Additional file 2: Table S2. Results of Cognitive correlates in relation to ADHD and high intelligence. [file 11689_2020_9307_MOESM2_ESM.docx]

**Cognitive correlates of Attention Deficit Hyperactivity Disorder in children and adolescents with high intellectual capacity.**

**Additional file 2: Cognitive correlates in relation to ADHD and high intelligence.**

Results regarding the cognitive correlates in Cohorts 1 and 2 are shown in Table S2.

**Table A2.** z-scores of the cognitive variables (higher scores indicating poorer performance) in the four groups, separately shown for cohort 1 and 2. Main effects of ADHD (yes/no) and IQ (average/high) and the interaction-effect were tested. Posthoc- t-test were carried out to compare the performance of highly intelligent individuals with ADHD compared to the typical (i.e. average intelligent) control group.

| ***COHORT 1*** | | | | | | | | |  |
| --- | --- | --- | --- | --- | --- | --- | --- | --- | --- |
|  | **z-score (SD)** | | | | | **ADHD** | **IQ** | **ADHD*IQ** | **Posthoc analysis** |
| **Task** | **ADHD**  **IQ≥120** | | **Control**  **IQ≥120** | **ADHD**  **IQ 90-110** | **Control**  **IQ 90-110** | ***F [d]*** | ***F [d]*** | ***F [d]*** | ***T-student [D]*** |
| **Inhibition** | | | | | | | | |  |
| Stop task-mean reaction time | -0.02[0.83] | | -0.21[0.91] | 0.18[0.81] | 0.01[0.99] | n.s | n.s | n.s | n.s. |
| Stop task-reaction time variability | 0.06[0.95] | | -0.27[0.80] | 0.37[0.85] | -0.20[0.86] | 13.40 [0.71] | n.s | n.s | n.s. |
| Stop task-stop signal reaction time | 0.27[0.94] | | -0.22[0.64] | 0.08[0.82] | -0.14[0.82] | 10.41 [0.63] | n.s. | n.s. | n.s. |
| **Verbal Working memory** | | | | | | | | |  |
| Digit span: maximum span forwards | 0.24[0.95] | | 0.18[0.77] | -0.24[0.87] | -0.14[1.03] | n.s | 9.74 [0.61] | n.s | n.s. |
| Digit span: maximum span backwards | 0.18[1.08] | | 0.26[0.72] | -0.32[0.79] | -0.06[1] | n.s | 10.82 [0.65] | n.s | n.s |
| **Timing variability** | | | | | | | | |  |
| Baseline speed left hand mean reaction time (ms) | -0.01[0.92] | | -0.30[0.96] | 0.00[0.91] | 0.00[0.76] | n.s | n.s | n.s | n.s |
| Baseline speed right hand mean reaction time (ms) | -0.04[0.90] | | -0.22[0.91] | 0.06[0.80] | -0.03[0.91] | n.s | n.s | n.s | n.s |
| **Motor coordination** | | | | | | | | |  |
| Pursuit: deviation left hand | -0.07[0.97] | | -0.14[0.80] | 0.01[0.88] | -0.01[0.88] | n.s | n.s | n.s | n.s |
| Pursuit: deviation right hand | -0.05[0.85] | | -0.19[0.91] | 0.02[0.89] | -0.09[0.94] | n.s | n.s | n.s | n.s |
| **Time estimation ability** | | | | | | | | |  |
| Time test: percentage absolute discrepancy 4; 8; 12; 16; 20 sec | -0.08[0.69] | | -0.21[0.66] | 0.24[0.75] | -0.28[0.57] | 8.11 [0.56] | n.s | n.s | n.s |
| **Motor speed** | | | | | | | | |  |
| Motor timing: median RT* | 0.00[0.91] | | 0.31[0.77] | -0.04[0.91] | -0.01[0.85] | n.s. | n.s. | n.s. | n.s |
| Motor timing: SD RT* | -0.01[0.94] | | -0.18[0.74] | 0.31[0.83] | -0.32[0.84] | 9.74 [0.61] | n.s. | n.s. | n.s |
| ***COHORT 2*** | | | | | | | | |  |
| **Timing variability** | | | | | | | | |  |
| Baseline speed left hand mean reaction time (ms) | | 0.14[0.94] | -0.22[1.02] | 0.18[0.75] | -0.11[1.07] | n.s. | n.s. | n.s. | n.s |
| Baseline speed right hand mean reaction time (ms) | | 0.08[0.82] | -0.42[0.78] | 0.33[0.84] | -0.38[1.22] | n.s. | n.s. | n.s. | n.s |
| **Sustained attention** | | | | | | | | |  |
| Sustained attention dots: mean reaction time hits | | 0.01[1] | -0.28[0.91] | 0.46[1.03] | -0.21[0.73] | n.s. | n.s. | n.s. | n.s |
| Sustained attention dots: Proportion misses | | 0.20[0.86] | -0.27[1.11] | 0.08[0.77] | 0.01[1.01] | n.s. | n.s. | n.s. | n.s |
| **Shifting Attentional set** | | | | | | | | |  |
| Shifting set: part 1 mean reaction time compatible | | -0.50[1.01] | -0.17[0.94] | -0.11[0.83] | 0.33[1.03] | n.s. | n.s. | n.s. | n.s |
| Shifting set: part 2 mean reaction time incompatible | | -0.35[0.94] | -0.16[0.76] | -0.35[0.94] | 0.54[1.13] | n.s. | 7.06 [0.80] | n.s. | -2.87 [-0.85] |
| Shifting set: part 3 mean reaction time compatible+incompatible | | -0.43[0.96] | -0.14[0.76] | 0.11[1.06] | 0.45[0.82] | n.s. | 8.65 [0.88] | n.s. | -3.27 [-0.98] |
| Shifting set: part 1 percentage errors compatible | | 0.10[0.98] | 0.03[0.77] | 0.01[0.80] | -0.26[0.92] | n.s. | n.s. | n.s. | n.s |
| Shifting set: part 2 percentage errors incompatible | | -0.26[0.86] | -0.22[0.79] | 0.45[1.01] | 0.18[0.84] | n.s. | 8.89 [0.90] | n.s. | n.s |
| Shifting set: part 3 percentage errors compatible+incompatible | | -0.17[0.93] | -0.37[0.78] | 0.14[0.93] | 0.46[1] | n.s. | 8.75 [0.89] | n.s. | n.s. |
| **Pattern recognition** | | | | | | | | |  |
| Feature identification: mean reaction time hits similar | | -0.31[0.92] | -0.53[0.76] | 0.67[0.87] | 0.13[0.86] | n.s. | 20.36 [1.35] | n.s. | n.s |
| Feature identification: mean reaction time hits dissimilar | | -0.30[0.76] | -0.47[0.97] | 0.44[0.99] | 0.28[0.82] | n.s. | 15.59 [1.19] | n.s. | n.s |
| **Working memory** | | | | | | | | |  |
| Memory search letters: mean reaction time hits average part 1+2+3 | | 0.03[1.18] | -0.47[0.78] | 0.14[0.91] | 0.28[0.80] | n.s. | n.s. | n.s. | n.s |
| Memory search letters: number of misses average part 1+2+3 | | 0.21[1.05] | 0.00[0.85] | -0.44[0.91] | 0.23[0.87] | n.s. | n.s. | n.s. | n.s |

**Note:***median RT=Median reaction time; *SD RT = Standard deviation of reaction time; effects are presented that survived correction for multiple testing using the False Discovery Rate procedure with a q-value setting of 0.05.
